# Prevalence, Predictors, and the Financial Impact of Missed Appointments in an Academic Adolescent Clinic

**DOI:** 10.7759/cureus.3613

**Published:** 2018-11-19

**Authors:** Justin D Triemstra, Lisa Lowery

**Affiliations:** 1 Pediatrics, Spectrum Health, Michigan State University, Grand Rapids, USA

**Keywords:** missed appointments, adolescent pediatrics, financial analysis

## Abstract

Introduction

Missed appointments have been shown to have significant economic consequences on clinics and health systems. Furthermore, adolescents have been shown to miss appointments more frequently than other pediatric patients. Information regarding predictive factors for which patients will miss appointments has been researched for adolescents, but scheduling-related risk factors and the financial impact of missed appointments in an adolescent clinic are lacking. Therefore, we sought to identify the financial impact and schedule-related risk factors of missed appointments in an academic adolescent clinic.

Methods

A retrospective chart review of financial and scheduling information of each missed appointment was conducted at the Adolescent and Young Adult Medicine Clinic at Helen DeVos Children’s Hospital (HDVCH) in Grand Rapids, Michigan between November 2016 and October 2017. Information obtained was the day of the week, time of appointment, lead days till appointment, level of provider, historical weather data, appointment type (new patient, established physical, established visit, or injection only) and insurance type for each patient.

Results

We report a 21.2% missed appointment rate. Monday had a significantly higher fraction of missed appointments than other days of the week (*p* < 0.001). An increase in nonattendance was also seen with an increase in the lead days until appointment (*p* = 0.026). Weather (*p* = 0.507), time of day (*p* = 0.665) and type of provider (*p* = 0.361) were not found to be significant. We estimated an annual billing and reimbursement loss from missed appointments of $170,100 and $51,289, respectively.

Conclusion

These results highlight that revenue lost from missed appointments is significant and directly affects the ability to improve patient access and care. Future efforts to decrease missed appointments should target schedule-related risk factors in addition to patient reminder systems.

## Introduction

Academic medical centers (AMC) are under significant financial stress [1]. According to recent reports of the Association of American Medical Centers, teaching hospitals represent only 5% of total hospitals and, however, provide up to one-third of all charity care and one-quarter of all Medicaid hospitalizations [1-2]. This imbalance directly contributes to lower operating margins and increased financial stress [2]. To counter these increased costs and decreased reimbursement rates, the AMCs must focus on operating efficiently to maintain the operating margins.

One major barrier to efficiency is missed appointments or no-shows. In the literature, a missed appointment is defined as a patient who misses a scheduled appointment or cancels within 24 hours of a scheduled appointment [3]. In addition to a missed opportunity to provide care to a patient, these missed appointments have significant economic consequences and contribute to the financial stress of AMCs [1,3]. In pediatrics, missed appointments have been studied broadly and reported to be greater than 20% of the scheduled visits, with higher rates reported in the adolescent population [4].

Adolescent providers have used different types of interventions to decrease patients' missed appointments. Previous studies have focused on identifying specific characteristics that are associated with increased missed appointment rates. These studies have shown that patient age, prior history of missed appointments, the month of appointment and the time of day have an increased risk of nonattendance [4-5]. Other studies have focused on the methods to decrease the nonattendance rate, which include patient reminder systems, targeted overbooking and penalization [6-7].

Although these methods have shown modest improvement, the rate and financial impact of nonattendance have continued to be substantial in subspecialty pediatric clinical settings [8-9]. Specific data regarding this impact in adolescent clinics, the most likely population to miss appointments in pediatrics, however, is lacking. Furthermore, although some information regarding predictive factors for which patients will miss appointments have been researched [4-5], scheduling-related risk factors are minimal in the adolescent literature. To address this gap, we investigated the financial impact and scheduling-related risk factors of missed appointments in an academic adolescent clinic.

## Materials and methods

The study was conducted at the Adolescent and Young Adult Medicine Clinic at Helen DeVos Children’s Hospital (HDVCH) in Grand Rapids, Michigan between November 2016 and October 2017 (2.5 provider full-time equivalent - FTE). This clinic's population results in an insurance payor mix of about 55% Medicaid, 41% private insurance and 4% other (self-pay, governmental insurance, etc.). A retrospective analysis using Epic (Verona, Wisconsin) electronic health record was completed, and 765 missed appointments were identified and reviewed. We defined a missed appointment as a visit-missed or same-day cancellation (<24 hours in advance). During the study period, the method to decrease missed appointments was automated phone call reminders and text message reminders (for Monday visits, the call was placed on the previous Friday). The missed appointment rate was calculated as the fraction of missed appointments to the number of all appointments [3]. Among the patients who missed appointments, we obtained information related to day of week, time of appointment, lead days till appointment, level of provider, historical weather data (AccuWeather; State College, Pennsylvania), appointment type (new patient, established physical, established visit, or injection only) and insurance type (Medicaid or commercial).

Linear regression was used to compare the time of appointment, lead days and the level of provider with the fraction of missed appointments. For these analyses, clustering on the day of the year was used to obtain robust standard errors to adjust for non-independent observations. An analysis of variance was conducted to compare the days of week and types of weather precipitation to the fraction of missed appointments. For the financial analysis, a senior financial analyst at our institution determined the average cost of encounter and the average cost of reimbursement for each type of visit and insurance at our clinic. The cost of each individual missed appointment was then coded by the type of visit and the insurance of the patient at the time of appointment and stored in Microsoft Excel (Redmond, Washington). Total yearly billing and reimbursement loss were determined by the summation of all missed appointments. The Spectrum Health Institutional Review Board deemed this study as not a human research and was exempt.

## Results

In our cohort of patients who missed appointments, we report a 21.2% missed appointment rate (clinical volume = 3,583; missed appointment = 760) during the study period. Monday had the statistically significant highest fraction of missed appointments compared to other days of the week (*p* < 0.001; Table 1). Also, there was a statistically significant increase in missed appointments with an increase in lead days from the appointment (*t* = 2.24, *p* = 0.026; Table 2). We found no significant difference between weather/precipitation (*F* = 0.78, *p* = 0.507), morning versus afternoon appointments (*t* = 0.43, *p* = 0.665) and the level of provider (midlevel provider vs physician; *t* = -0.92, *p* = 0.361; Table 2). After financial analysis, we estimated an annual billing loss of $170,100 and reimbursement loss of $51,289. On average, each missed appointment cost $292.70 of billing charges and a loss of $92.24 of reimbursement revenue.

**Table 1:** Fraction of missed appointments by day of week and its relationship with missed appointment rate a = ANOVA (analysis of variance)

Day of Week	Mean Fraction of Missed Appointments	Standard Deviation	ANOVA Analysis (F value)^a^	*p*-Value
Monday	0.26	0.13	5.42	<.001
Tuesday	0.24	0.15		
Wednesday	0.22	0.14		
Thursday	0.16	0.11		
Friday	0.17	0.09		

**Table 2:** Time of visit, level of provider and weather’s association with fraction of missed appointments per day a = Linear regression analysis; b = ANOVA (analysis of variance

	Observation (*n*)	Mean Fraction of Missed Appointments	Standard Error	Linear Regression or ANOVA	*p*-Value
Morning Appointment	252 (33.2%)	0.28	0.02	*t* = 0.43^a^	0.66
Afternoon Appointment	508 (66.8%)	0.29	0.01		
Physician	471 (62.0%)	0.29	0.01	*t* = -0.92^a^	0.36
Midlevel Provider	289 (38.0%)	0.27	0.02		
Fog	38	0.21	0.02	*F* = 0.78^b^	0.51
Rain	108	0.20	0.01		
Snow	44	0.20	0.02		
Thunderstorm	29	0.22	0.03		
Lead Days	760	Not applicable	0.04	*t* = 2.24	0.03

In our cohort of patients who missed appointments, 77% (*n *= 585) of our patients who missed appointments were publicly insured, 17.8% (*n *= 135) were commercially insured and 5.2% (*n *= 40) had no insurance. The vast majority of missed appointments were from established visits [established follow-up/acute visit = 74.3% (*n* = 565), established physical = 15.8% (*n *= 120), injection only = 5.6% (*n *= 42) and new patient visit = 4.3% (*n* = 33)].

## Discussion

In this study, we investigated the scheduling-related risk factors and the financial impact of missed appointments in a small academic adolescent clinic. This is one of the first studies to report financial data on the missed appointments for the adolescent patient population. Our data showed a substantial amount of the lost revenue from the missed appointments, a significant increase of nonattendance on days earlier in the week and an increase in missed appointment rate with an increase in lead days from the appointment.

Missed appointments continue to be a prevalent problem in pediatric populations [4-5,9-10]. Our data confirmed this finding in an academic adolescent clinic. Similar to the previously reported large-scale studies [10], our data indicated that Monday had the highest rate of nonattendance for adolescents (Table 1). We hypothesize that this could be related to the extended time between the appointment and the patient reminder calls (i.e. call placed on previous Friday instead of the day before the appointment) or more negative emotional feelings on Mondays than other days of the week leading to less motivation to attend a visit [10]. This finding is important to recognize prior to implementation of any intervention to decrease the no-show rate in an adolescent clinic.

Interestingly, we did not find that the time of day or the type of provider affected the missed appointment rate in our adolescent population (Table 2). This is different from other studies in adolescents [5]. Also, weather precipitation did not affect that appointment rate, which contradicts previously reported primary care provider perceptions on this phenomenon [11]. These findings may be limited by a single-site study design or geographical differences in patient populations when compared to other studies.

Our study also details a significant loss of revenue from a small academic adolescent clinic due to missed appointments. Adolescent primary-care clinics already face additional financial and efficiency challenges in comparison to other primary-care settings and missed appointments only contribute to this increased financial stress. Our study provides specific financial data that clinic administrators and adolescent providers can use to help plan future interventions to decrease the missed appointment rates in their clinical settings.

Limitations of this study include that data was obtained from a single institution in Grand Rapids, Michigan, United States and may affect the generalizability of our study. Second, certain demographics like gender, family income and distance from the clinic were not obtained, although this information has been reported previously in the adolescent literature. Third, our study did not include a control group of patients who did not miss appointments.

## Conclusions

In this study, we identified that the day of the week, increased lead days to appointment and a substantial financial impact are associated with the missed appointments in an academic adolescent clinic. This lost revenue from the missed appointments directly affects a clinic's financial ability to improve patient access and provide care for these vulnerable populations treated at AMCs. Utilizing operational strategies to maximize every possible dollar of revenue in academic adolescent clinics is essential to continue improving patient access and provide care in these important clinical settings.
